# Supplementary material for: The Effects of Chronic Unpredictable Mild Stress and Semi-Pure Diets on the Brain, Gut and Adrenal Medulla in C57BL6 Mice
Source: Int J Mol Sci. 2023 Sep 27;24(19):14618. doi: 10.3390/ijms241914618 (PMC10572190; doi:10.3390/ijms241914618)
Supplement: Supplementary file 1 [file ijms-24-14618-s001.zip › Supplementary Data.pdf]

Supplementary Data

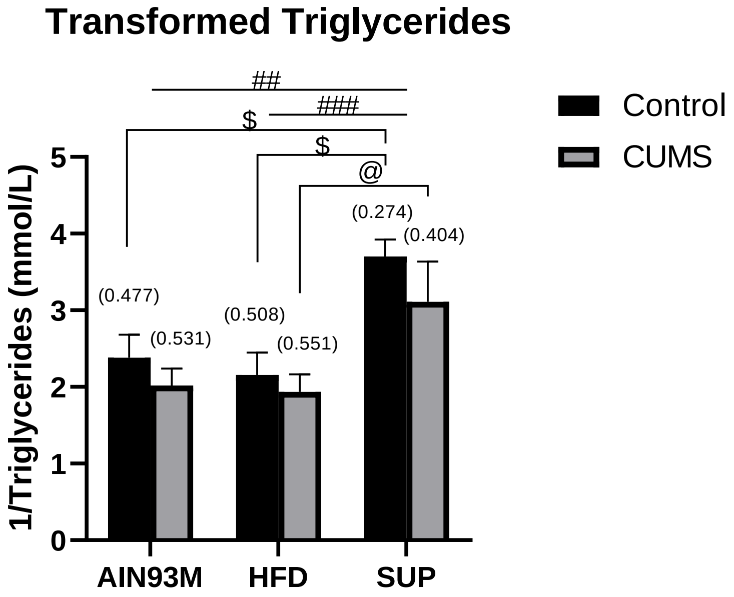

Figure S1. The effects of chronic unpredictable mild stress and diet on serum triglyceride levels. Two-way ANOVA with Bonferroni multiple comparisons. Data did not follow Gaussian distribution and was inversely transformed prior to analysis. Untransformed mean values are presented above the column for each group. $ Denotes a difference in control means between diets, $ = $p < 0.05$. @ Denotes a difference in CUMS means between diets, @ = $p < 0.05$. # Denotes a difference in main effect of diet, ## = $p < 0.01$, ### = $p < 0.001$. Control AIN93M $n = 6$, control HFD $n = 6$, control SUP $n = 6$, CUMS AIN93M $n = 6$, control HFD $n = 6$, control SUP $n = 6$. AIN93M, Semi-Pure Maintenance Diet; HFD, High Fat Diet; SUP, Superfood diet; CUMS, chronic unpredictable mild stress.

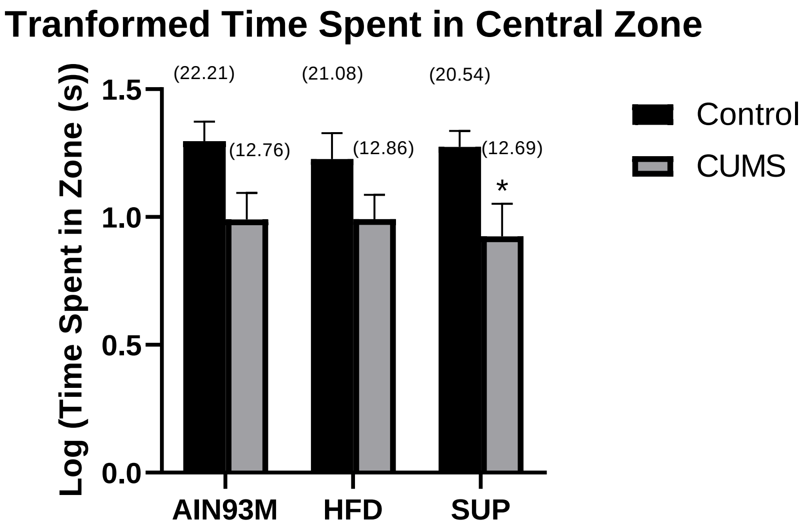

Figure S2. The effects of chronic unpredictable mild stress and diet on time spent in the central zone in the Open Field Test. Two-way ANOVA with Bonferroni multiple comparisons. Data did not follow Gaussian distribution and was logarithmically transformed prior to analysis. Untransformed mean values are presented above the

column for each group. * (On graph) Denotes a significant difference between control and CUMS means, * = $p < 0.05$. Control AIN93M n = 10, control HFD n = 10, control SUP n = 10, CUMS AIN93M n = 9, CUMS HFD n = 12, CUMS SUP n = 11. AIN93M, Semi-Pure Maintenance Diet; HFD, High Fat Diet; SUP, Superfood diet; CUMS, chronic unpredictable mild stress.

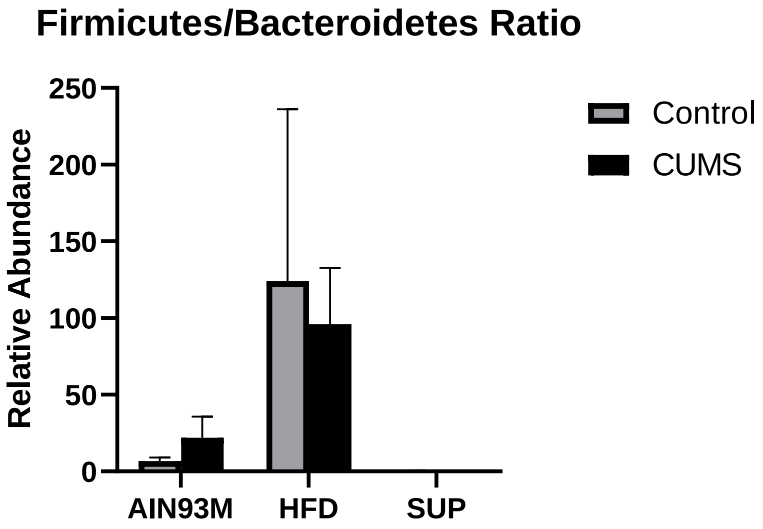

Figure S3. The effects of chronic unpredictable mild stress and diet on the Firmicutes to Bacteroidetes ratio of the relative abundance of gut bacterial phyla. Two-way ANOVA with Bonferroni multiple comparisons. N = 6 for all groups (samples combined in twos per group before analysis). AIN93M, Semi-Pure Maintenance Diet; HFD, High Fat Diet; SUP, Superfood diet; CUMS, chronic unpredictable mild stress.
