# Supplementary material for: The Effects of Chronic Unpredictable Mild Stress and Semi-Pure Diets on the Brain, Gut and Adrenal Medulla in C57BL6 Mice
Source: Int J Mol Sci. 2023 Sep 27;24(19):14618. doi: 10.3390/ijms241914618 (PMC10572190; doi:10.3390/ijms241914618)
Supplement: Supplementary file 1 [file ijms-24-14618-s001.zip › Supplementary Diet Data.pdf]

Specialty Feeds

3150 Great Eastern Hwy
Glen Forrest
Western Australia 6071
p: +61 8 9298 8111
F: +61 8 9298 8700
Email: info@specialtyfeeds.com

Diet

SF08-020

Standard AIN93M Rodent Diet Plus Extra Vitamins

A semi-pure diet formulation for laboratory rats and mice based on AIN-93M with extra vitamins.

- We have become increasingly concerned about the effects of irradiation on vitamins in diets based on AIN93. We have some evidence of an apparent deficiency in pregnant rats and mice when these diets have been irradiated at 25 KGy. We have been able to overcome this problem by increasing the vitamin inclusion rates.

Calculated Nutritional Parameters as Fed	
Protein	13.6%
Total Fat	4.0%
Total Digestible Carbohydrate as defined by FSANZ standard 1.2.8	64.3%
Crude Fibre	4.7%
AD Fibre	4.7%
Digestible Energy	15.5 MJ / Kg
Net Metabolisable Energy	13.7 MJ / Kg
% Total calculated digestible energy from lipids	9.0%
% Total calculated Net Metabolisable Energy from Lipids	10.7%
% Total calculated digestible energy from protein	15.0%
% Total calculated Net Metabolisable Energy from Protein	13.8%

Diet Form and Features

- Semi pure diet. 12 mm diameter pellets.
- Pack size 5 Kg, vacuum packed in oxygen impermeable plastic bags, under nitrogen. Bags are packed into cardboard cartons for protection during transit. Smaller pack quantity on request.
- Diet suitable for irradiation but not suitable for autoclave.
- Lead time 2 weeks for non-irradiation or 4 weeks for irradiation.

ISO 9001 certified Equal Assurance

FEEDSAFE ACCREDITED FEED MANUFACTURER

JAS-ANZ

AUSTRALIAN TRUSTED TRADER INTERNATIONAL AEO AND TRADE FACILITATION

Ingredients	
Casein (Acid)	140 g/Kg
Sucrose	100 g/Kg
Canola Oil	40 g/Kg
Cellulose	50 g/Kg
Wheat Starch	472 g/Kg
Dextrinised Starch	155 g/Kg
L Methionine	1.8 g/Kg
Calcium Carbonate	13.1 g/Kg
Sodium Chloride	2.6 g/Kg
AIN93 Trace Minerals	1.4 g/Kg
Potassium Citrate	1.0 g/Kg
Potassium Dihydrogen Phosphate	8.8 g/Kg
Potassium Sulphate	1.6 g/Kg
Choline Chloride (75%)	2.5 g/Kg
AIN93 Vitamins	15 g/Kg
Vitamin K (0.23%)	0.9 g/Kg

Calculated Amino Acids as Fed	
Valine	0.90%
Leucine	1.30%
Isoleucine	0.60%
Threonine	0.60%
Methionine	0.60%
Cysteine	0.05%
Lysine	1.00%
Phenylalanine	0.70%
Tyrosine	0.70%
Histidine	0.42%
Tryptophan	0.20%

Calculated Total Minerals as Fed	
Calcium	0.47%
Phosphorous	0.35%
Magnesium	0.09%
Sodium	0.15%
Chloride	0.16%
Potassium	0.40%
Sulphur	0.17%
Iron	75 mg/Kg
Copper	6.9 mg/Kg
Iodine	0.2 mg/Kg
Manganese	19.5 mg/Kg
Cobalt	No data
Zinc	47 mg/Kg
Molybdenum	0.15 mg/Kg
Selenium	0.3 mg/Kg
Cadmium	No data
Chromium	1.0 mg/Kg
Fluoride	1.0 mg/Kg
Lithium	0.1 mg/Kg
Boron	3.1 mg/Kg
Nickel	0.5 mg/Kg
Vanadium	0.1 mg/Kg

Calculated Total Vitamins as Fed	
Vitamin A (Retinol)	6 000 IU/Kg
Vitamin D (Cholecalciferol)	1 500 IU/Kg
Vitamin E (a Tocopherol acetate)	114 mg/Kg
Vitamin K (Menadione)	3.5 mg/Kg
Vitamin C (Ascorbic acid)	None added
Vitamin B1 (Thiamine)	9 mg/Kg
Vitamin B2 (Riboflavin)	9.2 mg/Kg
Niacin (Nicotinic acid)	45 mg/Kg
Vitamin B6 (Pryridoxine)	10.6 mg/Kg
Pantothenic Acid	24 mg/Kg
Biotin	300 ug/Kg
Folic Acid	3 mg/Kg
Inositol	None added
Vitamin B12 (Cyancobalamin)	152 ug/Kg
Choline	1 450 mg/Kg

Calculated Fatty Acid Composition as Fed	
Myristic Acid 14:0	No data
Palmitic Acid 16:0	0.20%
Stearic Acid 18:0	0.10%
Palmitoleic Acid 16:1	No data
Oleic Acid 18:1	2.40%
Gadoleic Acid 20:1	Trace
Linoleic Acid 18:2 n6	0.80%
a Linolenic Acid 18:3 n3	0.56%
Arachadonic Acid 20:4 n6	No data
EPA 20:5 n3	No data
DHA 22:6 n3	No data
Total n3	0.56%
Total n6	0.86%
Total Mono Unsaturated Fats	2.28%
Total Poly Unsaturated Fats	1.43%
Total Saturated Fats	0.29%

Calculated data uses information from typical raw material composition. It could be expected that individual batches of diet will vary from this figure. **Diet post treatment by irradiation or autoclave could change these parameters**. We are happy to provide full calculated nutritional information for all of our products, however we would like to emphasise that these diets have been specifically designed for manufacture by Specialty Feeds.

3150 Great Eastern Hwy
Glen Forrest
Western Australia 6071
p: +61 8 9298 8111
F: +61 8 9298 8700
Email: info@specialtyfeeds.com

Diet

SF03-030 23% Fat, High Glycaemic Index Semi-Pure Rodent Diet

A semi-pure diet formulation for laboratory rats and mice based on AIN-93G. This formulation has been designed to maximise the risk of developing type II diabetes.

- The only carbohydrates present are cellulose, as a fibre source, and dextrose. Theoretically the glycaemic index of this diet should be close to 100.

Calculated Nutritional Parameters

Protein	19.40%
Total Fat	23.00%
Crude Fibre	4.66%
AD Fibre	4.66%
Digestible Energy	20 MJ / Kg
% Total calculated digestible energy from lipids	42.00%
% Total calculated digestible energy from protein	17.00%

Diet Form and Features

- Semi pure diet. 12 mm diameter pellets.
- Pack size 1.5 Kg, vacuum packed in oxygen- impermeable plastic bags, under nitrogen. Bags are packed into cardboard cartons to protect them during transit. Smaller pack quantity on request.
- Diet suitable for irradiation but not suitable for autoclave. Note, Irradiation can soften pellets.
- Lead time 2 weeks for non-irradiation or 4 weeks for irradiation.

Ingredients

Casein (Acid)	200 g/Kg
Dextrose	505 g/Kg
Canola Oil	50 g/Kg
Cocoa Butter	50 g/Kg
Hydrogenated Vegetable Oil (Copha)	131 g/Kg
Cellulose	20 g/Kg
DL Methionine	3.0 g/Kg
Calcium Carbonate	13.1 g/Kg
Sodium Chloride	2.6 g/Kg
AIN93 Trace Minerals	1.4 g/Kg
Potassium Citrate	2.5 g/Kg
Potassium Dihydrogen Phosphate	6.9 g/Kg
Potassium Sulphate	1.6 g/Kg
Choline Chloride (75%)	2.5 g/Kg
AIN93 Vitamins	10 g/Kg

Calculated Amino Acids

Valine	1.26%
Leucine	1.80%
Isoleucine	0.90%
Threonine	0.80%
Methionine	0.80%
Cystine	0.06%
Lysine	1.50%
Phenylanine	1.00%
Tyrosine	1.00%
Histidine	0.60%
Tryptophan	0.30%

Calculated Total Minerals

Calcium	0.47%
Phosphorous	0.32%
Magnesium	0.09%
Sodium	0.12%
Chloride	0.16%
Potassium	0.40%
Sulphur	0.20%
Iron	72 mg/Kg
Copper	7 mg/Kg
Iodine	0.2 mg/Kg
Manganese	18 mg/Kg
Cobalt	No data
Zinc	52 mg/Kg
Molybdenum	0.15 mg/Kg
Selenium	0.3 mg/Kg
Cadmium	No data
Chromium	1.0 mg/Kg
Fluoride	1.0 mg/Kg
Lithium	0.1 mg/Kg
Boron	2.1 mg/Kg
Nickel	0.5 mg/Kg
Vanadium	0.1 mg/Kg

Calculated Total Vitamins

Vitamin A (Retinol)	4 000 IU/Kg
Vitamin D (Cholecalciferol)	1 000 IU/Kg
Vitamin E (a Tocopherol acetate)	78 mg/Kg
Vitamin K (Menadione)	1 mg/Kg
Vitamin C (Ascorbic acid)	None added
Vitamin B1 (Thiamine)	6.1 mg/Kg
Vitamin B2 (Riboflavin)	6.3 mg/Kg
Niacin (Nicotinic acid)	30 mg/Kg
Vitamin B6 (Pryridoxine)	7.2 mg/Kg
Pantothenic Acid	16.5 mg/Kg
Biotin	200 ug/Kg
Folic Acid	2 mg/Kg
Inositol	None added
Vitamin B12 (Cyancobalamin)	103 ug/Kg
Choline	1670 mg/Kg

Calculated Fatty Acid Composition

Saturated fats C12 or Less	6.80%
Myristic Acid 14:0	1.80%
Palmitic Acid 16:0	3.20%
Stearic Acid 18:0	3.10%
Oleic Acid 18:1	5.92%
Gadoleic Acid 20:1	0.10%
Linoleic Acid 18:2 n6	1.40%
a Linolenic Acid 18:3 n3	0.60%
Stearidonic Acid 18:4 n3	0.03%
EPA 20:5 n3	Trace
DHA 22:6 n3	No data
Total n3	0.60%
Total n6	1.40%
Total Saturated Fats	14.90%
Total Monosaturated Fats	6.10%
Total Polyunsaturated Fat	2.00%
Cholesterol	No data

Calculated data uses information from typical raw material composition. It could be expected that individual batches of diet will vary from this figure. **Diet post treatment by irradiation or auto clave could change these parameters**. We are happy to provide full calculated nutritional information for all of our products, however we would like to emphasise that these diets have been specifically designed for manufacture by Specialty Feeds.

Specialty Feeds

3150 Great Eastern Hwy
Glen Forrest
Western Australia 6071
p: +61 8 9298 8111
F: +61 8 9298 8700
Email: info@specialtyfeeds.com

Diet

SF21-202 High Fibre, Fruit and Vegetable Powder Modification of AIN93M For Irradiation

A semi-pure diet formulation for laboratory rats and mice based on AIN-93M. This formulation satisfies the maintenance nutritional requirements of rats and mice. Some modifications have been made to the original formulation to suit locally available raw materials.

- Kale powder, pumpkin powder, spinach leaf powder, mixed berry powder, carrot juice powder and beetroot juice powder have all been added at 2% w/w
- Cacao powder has been added at 8% w/w
- Cellulose has been increased to 10% w/w, psyllium husk has been added at 10% w/w and guar gum has been added at 20% w/w.
- Casein, Canola Oil, Sucrose, Dextrinised Starch and Wheat starch have all been reduced.
- Complete nutrient data is incomplete for Kale, pumpkin, spinach leaf, mixed berry, carrot juice, beetroot juice and cacao powder. Total calculated amino acids, minerals, vitamins and fatty acids will be under reported due to this.
- Vitamins have been increased for irradiation

Calculated Nutritional Parameters as Fed	
Protein	13.8%
Total Fat	4.0%
Total digestible carbohydrate as defined by FSANZ Standard 1.2.8	32.5%
Crude Fibre	37.9%
AD Fibre	37.9%
Digestible Energy	9.9 MJ / Kg
% Total calculated digestible energy from lipids	14.8%
% Total calculated digestible energy from protein	22.7%

Diet Form and Features

- Semi pure diet. 12 mm diameter pellets.
- Pack size 5 Kg, vacuum packed in oxygen impermeable plastic bags, under nitrogen. Bags are packed into cardboard cartons for protection during transit. Smaller pack quantity on request.
- Diet suitable for irradiation but not suitable for autoclave.
- Lead time 2 weeks for non-irradiation or 8 weeks for irradiation.

ISO 9001 certified Equal Assurance
FEEDSAFE ACCREDITED FEED MANUFACTURER
JAS-ANZ
AUSTRALIAN TRUSTED TRADER INTERNATIONAL AEO AND TRADE FACILITATION

Ingredients	
Casein (Acid)	96 g/Kg
Sucrose	50 g/Kg
Canola Oil	29 g/Kg
Cellulose	100 g/Kg
Psyllium Husk	100 g/Kg
Guar Gum	200 g/Kg
Wheat Starch	230 g/Kg
Dextrinised Starch	75 g/Kg
Beetroot Juice Powder	11 g/Kg
Mixed Berry Powder	11 g/Kg
Cacao Powder	80 g/Kg
Carrot Juice Powder	11 g/Kg
Kale Powder	11 g/Kg
Pumpkin Powder	11 g/Kg
Spinack Leaf Powder	11 g/Kg
L Methionine	1.8 g/Kg
Calcium Carbonate	13.1 g/Kg
Sodium Chloride	2.6 g/Kg
AIN93 Trace Minerals	1.4 g/Kg
Potassium Citrate	1.0 g/Kg
Potassium Dihydrogen Phosphate	8.8 g/Kg
Potassium Sulphate	1.6 g/Kg
Choline Chloride (75%)	2.5 g/Kg
AIN93 Vitamins	15 g/Kg
Vitamin K 0.23% Active	0.87 g/Kg

Calculated Essential Amino Acids as Fed	
Valine	0.63%
Leucine	0.90%
Isoleucine	0.51%
Threonine	0.41%
Methionine	0.48%
Cysteine	0.05%
Lysine	0.79%
Phenylalanine	0.50%
Tyrosine	0.56%
Tryptophan	0.12%
Histidine	0.27%

Calculated Total Minerals as Fed	
Calcium	0.71%
Phosphorous	0.30%
Magnesium	0.06%
Sodium	0.14%
Chloride	0.16%
Potassium	0.55%
Sulphur	0.14%
Iron	49 mg/Kg
Copper	7.0 mg/Kg
Iodine	0.2 mg/Kg
Manganese	15 mg/Kg
Cobalt	No data
Zinc	42 mg/Kg
Molybdenum	0.15 mg/Kg
Selenium	0.2 mg/Kg
Cadmium	No data
Chromium	1.0 mg/Kg
Fluoride	1.0 mg/Kg
Lithium	0.1 mg/Kg
Boron	1.1 mg/Kg
Nickel	0.5 mg/Kg
Vanadium	0.1 mg/Kg

ISO 9001 certified
Equal Assurance
FEEDSAFE ACCREDITED
FEED MANUFACTURER
JAS-ANZ
AUSTRALIAN TRUSTED TRADER
INTERNATIONAL AEO AND TRADE FACILITATION

Calculated Total Vitamins as Fed	
Vitamin A (Retinol)	6 000 IU/Kg
Vitamin D (Cholecalciferol)	1 500 IU/Kg
Vitamin E (a Tocopherol acetate)	114 mg/Kg
Vitamin K (Menadione)	3.5 mg/Kg
Vitamin C (Ascorbic acid)	None added
Vitamin B1 (Thiamine)	9 mg/Kg
Vitamin B2 (Riboflavin)	9.2 mg/Kg
Niacin (Nicotinic acid)	46 mg/Kg
Vitamin B6 (Pryridoxine)	10.6 mg/Kg
Pantothenic Acid	24 mg/Kg
Biotin	300 ug/Kg
Folic Acid	3 mg/Kg
Inositol	None added
Vitamin B12 (Cyancobalamin)	150 ug/Kg
Choline	2 010 mg/Kg

Calculated Fatty Acid Composition as Fed	
Myristic Acid 14:0	Trace
Palmitic Acid 16:0	0.12%
Stearic Acid 18:0	0.06%
Palmitoleic Acid 16:1	Trace
Oleic Acid 18:1	1.60%
Gadoleic Acid 20:1	0.03%
Linoleic Acid 18:2 n6	0.63%
a Linolenic Acid 18:3 n3	0.40%
Arachadonic Acid 20:4 n6	No data
EPA 20:5 n3	No data
DHA 22:6 n3	No data
Total n3	0.40%
Total n6	0.63%
Total Mono Unsaturated Fats	1.64%
Total Polyunsaturated Fats	1.04%
Total Saturated Fats	0.20%

Calculated data uses information from typical raw material composition. It could be expected that individual batches of diet will vary from this figure. **Diet post treatment by irradiation or auto clave could change these parameters**. We are happy to provide full calculated nutritional information for all of our products, however we would like to emphasise that these diets have been specifically designed for manufacture by Specialty Feeds.

ISO 9001 certified
Equal Assurance
FEEDSAFE ACCREDITED FEED MANUFACTURER
AUSTRALIAN TRUSTED TRADER
INTERNATIONAL AEO AND TRADE FACILITATION
JAS-ANZ
